# Fever dreams: demons and nightmares in an ICU bed

**DOI:** 10.1007/s00134-026-08398-2

**Published:** 2026-03-26

**Authors:** Matthew F. Mart, Yahya Shehabi

**Affiliations:** 1 Division of Allergy, Pulmonary, and Critical Care Medicine, Department of Medicine, Vanderbilt University Medical Center, Nashville, USA; 2 Critical Illness, Brain Dysfunction, and Survivorship (CIBS) Center, Nashville, USA; 3 VA Tennessee Valley Healthcare System Geriatric Research Education and Clinical Center (GRECC), Nashville, USA; 4 Department of Intensive Care, School of Clinical Sciences, Monash Health, Victorian Heart Hospital, Monash University, Melbourne, Australia; 5 The Prince of Wales School of Medicine, University of New South Wales, Sydney, Australia

Wrought with night sweats and the vivid machinations of a brain on fire, a 70-year-old male with a history of diabetes and coronary artery disease presents to the emergency department with fevers and progressive hypoxemia requiring supplemental oxygen. He is confused and not oriented to time or place. Upon testing, he is diagnosed with influenza A, and his chest X-ray has bilateral infiltrates. Due to the severity of his illness, he is admitted to the intensive care unit (ICU). Over the next 24 h, he is intubated and mechanically ventilated. He is deeply sedated with propofol and fentanyl. He experiences intermittent confusion and significant nighttime agitation mixed with episodes of complete somnolence and limited interaction. These symptoms last for several days, for which he is treated with haloperidol. Approximately 1 week into his hospital course, he is extubated. He has occupational and physical therapy ordered, though his rehabilitation sessions are limited by ongoing drowsiness, distressing hallucinations, and difficulty remaining alert. He ultimately survives and is discharged home. In follow-up with his primary care physician, he notes difficulties with recalling appointments, focusing on tasks, and reports short-term amnesia and fading memory.

## Delirium: the brain on fire

Delirium is a form of acute neurologic organ failure manifesting as an acute change in attention that fluctuates over time with impairments in cognition, such as memory, orientation, or perception [1]. Importantly, delirium cannot be explained by another neurocognitive disorder and is triggered by an underlying disease, medications, or other substances. Delirium is extremely common [2, 3], as seen in our fictional patient case, where it can have wide-ranging psychomotor manifestations from severely depressed consciousness (i.e., hypoactive delirium) to significant agitation (i.e., hyperactive delirium) with patients reporting visual, auditory, and perceptional disturbances [4]. In the ICU, it is estimated that over half of mechanically ventilated adults may experience delirium, with hypoactive and mixed delirium being most common [2–4]. Delirium is a key predictor, independent of severity of illness, of poor outcomes in the critically ill including prolonged ventilation and increased mortality, yet delirium is frequently underrecognized by bedside clinicians [5]. For those reasons, routine screening using validated tools, such as the Confusion Assessment Method for the ICU (CAM-ICU) or the Intensive Care Delirium Screening Checklist (ICDSC), is recommended by international guidelines with the goal of recognizing and treating any modifiable causes with the goal of reducing delirium duration [2–4, 6]. Finally, among survivors, delirium is independently associated with long-term cognitive impairment, with up to one-quarter of survivors experiencing cognitive impairment of similar severity to that of mild Alzheimer’s disease or moderate traumatic brain injury out to 12 months with increased incident dementia diagnoses in the decade following delirium [2, 4].

## Pathogenesis and risk factors

The pathogenesis of delirium is complex and multi-faceted with several biological mechanisms likely involved. Inflammation, imbalances in neurotransmitter signaling, and deranged energy metabolism are frequently cited mechanisms [4]. Severe systemic inflammation leading to neuroinflammation, such as in our patient with septic shock due to influenza, is a strong trigger for delirium [7]. Similarly, imbalances in neurotransmitters, such as acetylcholine and dopamine, can occur during critical illness and may be associated with delirium [4]. Finally, dysregulated glucose metabolism and oxygen utilization may contribute to the development of delirium. Critically ill patients may have evidence of ischemia and microvascular dysfunction in the brain, and reduced neuronal energy substrate may contribute to the development of delirium [4]. Patients with underlying vulnerabilities (e.g., older age with comorbidities or pre-existing neurocognitive deficits) frequently suffer from baseline chronic neuroinflammation, dysregulated metabolism, and neurotransmitter imbalances due to chronic disease and pathological aging; when faced with the deranged physiology of critical illness, such patients have an even greater risk of delirium [4].

**Fig. 1:**
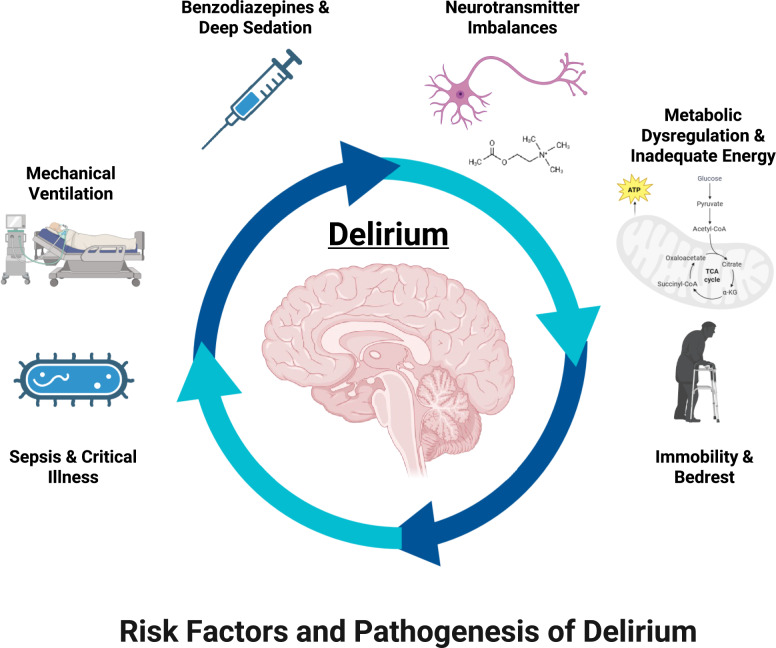
Risk factors and management of delirium

Care practices intrinsic to the ICU may contribute to the pathophysiology of delirium. The use of sedation and other centrally acting medications, especially benzodiazepines, combined with physical immobility, in concert with the inciting illness (e.g., sepsis, trauma, etc.), substantially increases the risk of delirium, which has been hypothesized to be due to changes in the blood–brain barrier, oxygen delivery, and increased neuroinflammation [4, 8, 9]. Deep sedation, including in the first 48 h as our patient received, is associated with increased delirium in addition to mortality [10]. Use of benzodiazepine-based sedation is one of the most significant predictors of developing delirium [11]. Additionally, the use of restraints, pain, and sleep deprivation all frequently occur in the ICU and may contribute to delirium by disrupting neuronal signaling that is already impaired in the context of pre-existing vulnerabilities and critical illness [4] (Fig. 1).

## Management

The multi-faceted etiology of delirium has hampered both prevention and treatment. Treatments focused on a singular mechanism, such as neurotransmitter imbalance, have been ineffective to date, such that current interventions predominantly focus on prevention and mitigation of delirium before it has developed. Intervention approaches that have been studied to date have included both non-pharmacological and pharmacological approaches. Non-pharmacological interventions such as early mobilization in the ICU, including performance of functional tasks related to activities of daily living, are associated with less delirium in critically ill medical and surgical patients [9, 12]. Pairing physical activity, which is associated with reduced inflammation, with cognitive tasks as required in daily activities, may improve cognitive processing and thereby reduce delirium [9]. International guidelines now recommend multidomain bundles of care prioritizing light sedation with daily sedation interruptions, avoidance of benzodiazepines, routine delirium screening, and early rehabilitation in the ICU to prevent or reduce the duration and severity of delirium in the ICU [13]. Additionally, early rehabilitation in the ICU may prevent long-term cognitive complications [9], raising the hope that the long-term impact of ICU delirium can at least be mitigated with best practices, even if they are not a definitive treatment.

Pharmacological management of delirium has been inconclusive. Antipsychotics such as haloperidol are commonly used to treat delirium based on mechanistic hypotheses regarding neurotransmitter imbalances, yet multiple randomized clinical trials (RCTs) have shown no benefit in the treatment of delirium [14–16]. Multiple RCTs have also evaluated sedation strategies and their impact on delirium in mechanically ventilated adults. The MENDS2 trial found no difference in delirium with dexmedetomidine vs. propofol in septic patients, while the SPICE III trial found that the dexmedetomidine group had a modestly greater number of days free from delirium and/or coma (i.e., less delirium and coma) (adjusted risk difference in days [95% CI] 1.0 [0.5–1.5]) [8, 17]. A recent practice recommendation, based on meta-analysis, reported 33% relative risk reduction with dexmedetomidine compared with other sedatives [18]. Low-dose nighttime infusion of dexmedetomidine may also reduce the incidence of delirium [19]. Finally, emerging and mostly experimental pharmacological approaches for delirium prevention, such as inhaled insulin, are being studied with potential promise in small phase II trials of surgical patients but remain investigational [20]. The quest for effective pharmacological delirium prevention and treatments is a research priority, which, in combination with non-pharmacological strategies, may conquer the multifaceted pathophysiology of delirium.

## Conclusions

Delirium is an acquired form of neurological organ failure common in the ICU. It leads to significant downstream complications, including mortality and long-term cognitive impairment. Its pathogenesis is complex, making prevention and treatment challenging. Non-pharmacological management mitigates delirium but does not eliminate the incidence of delirium. Key questions remain unanswered and represent crucial future research directions, including improved characterization of delirium mechanisms, development of targeted pharmacological treatments, and elucidation of the underlying pathophysiology of long-term cognitive impairment among ICU survivors.

## Data Availability

Not applicable.
